# Anoctamin 5 promotes osteosarcoma development by increasing degradation of Nel-like proteins 1 and 2

**DOI:** 10.18632/aging.203212

**Published:** 2021-07-09

**Authors:** Runsang Pan, Qiaoying Lu, Chong Ren, Hao Li, Fanqiang Zeng, Xiaobin Tian, Houping Chen

**Affiliations:** 1 Department of Orthopedics, Guiyang Maternal and Child Health-Care Hospital, Guiyang 550009, Guizhou, China; 2 Department of Orthopedics, The Affiliated Hospital of Guizhou Medical University, Guiyang 550009, Guizhou, China

**Keywords:** osteosarcoma, ANO5, NELL1, NELL2

## Abstract

Anoctamin 5 (ANO5) is a member of the Anoctamin (ANO) family of calcium-activated chloride channels. Although ANO5 expression is upregulated in various cancers, its role in osteosarcoma remains largely unknown. In this study, bioinformatics analysis, western blot, and immunohistochemical staining revealed that ANO5 was upregulated in osteosarcoma cell lines and osteosarcoma tissues, and ANO5 expression was positively associated with tumor size, tumor grade, and metastasis. Functional experiments demonstrated that inhibition of ANO5 decreased, while ANO5 overexpression increased, osteosarcoma cell proliferation and mobility *in vitro*. Immunoprecipitation, western blot, and confocal microscopy experiments showed that ANO5 bound to and promoted the degradation of Nel-like proteins 1 (NELL1) and 2 (NELL2). Moreover, a subcutaneous tumor transplantation model revealed that ANO5 knockdown reduced osteosarcoma cell proliferation and increased NELL1 and NELL2 expression *in vivo*. Finally, rescue experiments showed that knockdown of NELL1 or NELL2 reversed the inhibitory effects of ANO5 knockdown on osteosarcoma cell proliferation and migration. These results demonstrated that upregulation of ANO5 promoted osteosarcoma development by decreasing the stability of the NELL1 and NELL2 proteins and that ANO5 may be an effective target for the treatment of osteosarcoma.

## INTRODUCTION

Osteosarcoma is a common and very aggressive type of bone malignancy [1]. Despite advances in diagnosis and therapy, the overall prognosis of osteosarcoma patients remains poor. Moreover, recurrent metastatic osteosarcoma is observed in more than 80% of patients after surgery [2, 3]. It is therefore urgent to identify regulators of osteosarcoma progression in order to improve therapies for this type of cancer.

The Anoctamin (ANO) family, also called the transmembrane member 16 (TMEM16) family, is a class of calcium-activated chloride channels that regulate various biological functions, including ion transport and maintenance of membrane proteins [4, 5]. ANO family members are up-regulated in and promote the development of cancers. For example, ANO1 levels were increased in gastric cancer tissues and were positively associated with TNM stage [6]. Additionally, esophageal squamous cell carcinoma patients with higher ANO1 levels had poorer prognoses [7]. ANO9 was highly expressed in pancreatic cancer and promoted the metastasis of pancreatic cancer cells by increasing epidermal growth factor receptor expression [8]. Finally, ANO6 activated the ERK pathway and promoted glioma development [9].

Neural epidermal growth factor-like (Nel) proteins are a class of glycoproteins that are very similar in structure to thrombospondin1 and are secreted into the extracellular matrix. At present, two Nel family members, NELL1 and NELL2, have been identified in mammals [10]. NELL1 and NELL2 show greater than 50% homology in their amino acid sequences and map to chromosomes 11p15.1 and 12q12, respectively [11]. Physiologically, NELL1 and NELL2 promote differentiation during osteogenesis and osteocyte proliferation [12]. NELL1 expression is typically reduced in osteosarcoma compared with benign tumor tissues, indicating that it may act as a tumor suppressor [13]. However, little is known about the regulatory mechanisms associated with NELL proteins.

In this study, we found that ANO family member ANO5 was highly expressed in osteosarcoma tissues and cell lines and that it promoted the proliferation and metastasis of osteosarcoma cells by increasing the degradation of NELL1 and NELL2.

## RESULTS

### ANO5 was upregulated in osteosarcoma

**Figure 1:**
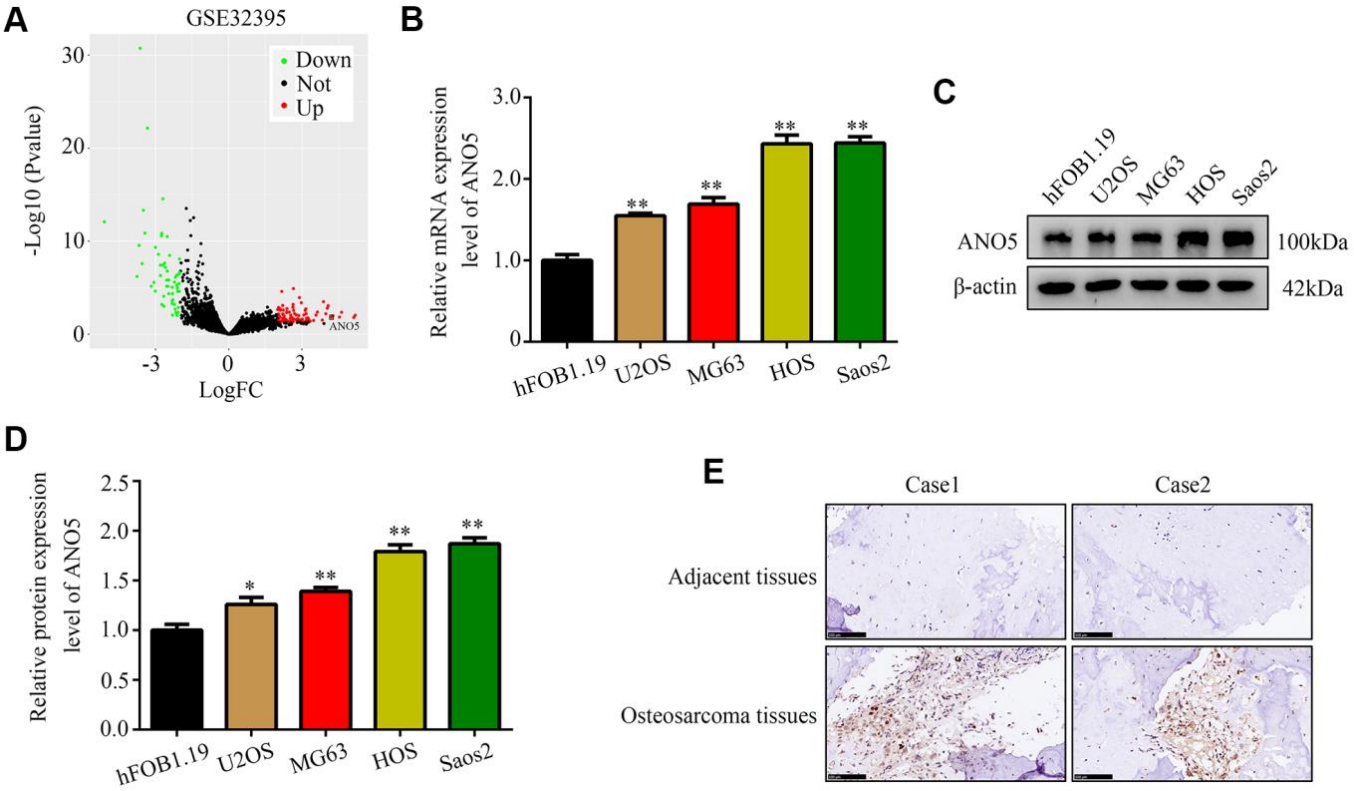
**ANO5 was upregulated in osteosarcoma.** (**A**) A volcano plot showed that the ANO5 gene was significantly upregulated in osteosarcoma cells. (**B**) qRT-PCR indicated that ANO5 was upregulated in osteosarcoma cells (U2OS, MG63, HOS, and Saos2) compared to the normal osteoblast cell line hFOB 1.19. (**C**, **D**) Western blot indicated that ANO5 was upregulated in osteosarcoma cells (U2OS, MG63, HOS, and Saos2) compared to the normal osteoblast cell line hFOB 1.19. (**E**) IHC demonstrated that ANO5 was more highly expressed in osteosarcoma tissues than in adjacent normal tissues. *, *P*<0.05; **, *P*<0.01.

First, we analyzed gene expression in the GSE32395 dataset, which contains the normal osteoblast cell line hFOB 1.19, the normal bone marrow stromal cell line L87/4, and seven osteosarcoma cell lines (HOS, HOS-58, U2OS, Saos2, MNNG, SJSA, and MG63). The results showed that ANO5 gene expression was markedly upregulated in osteosarcoma cell lines (Figure 1A). Using qRT-PCR and western blot, we found that ANO5 mRNA and protein levels were both elevated in osteosarcoma cells (U2OS, MG63, HOS, and Saos2) compared to the normal osteoblast cell line hFOB 1.19 (Figure 1B–1D). Furthermore, immunohistochemical staining of ANO5 in 40 paired osteosarcoma and adjacent normal tissues indicated that ANO5 was also elevated in osteosarcoma tissues (Figure 1E). Finally, we found that high ANO5 expression was positively associated with tumor size, tumor grade, and metastasis (Table 1). Together, these results indicate that ANO5 may act as an oncogene in osteosarcoma.

**Table 1:** Relationships between ANO5 expression and age, gender, tumor size, tumor grade, and metastasis in osteosarcoma patients were evaluated using a Chi-square test.

**Parameters**	**ANO5 expression**		**P value**
	**High**	**Low**	
Gender			0.996
Male	13	14	
Female	7	6	
Age			0.995
>19	6	5	
≤19	14	15	
Tumor size (cm)			0.022
>5	16	8	
≤5	4	12	
Tumor stage			0.019
I + IIA	3	11	
IIB/III	17	9	
Metastasis			0.025
Yes	15	7	
No	5	13	

### Inhibition of ANO5 reduced osteosarcoma cell proliferation and metastasis *in vitro*

**Figure 2:**
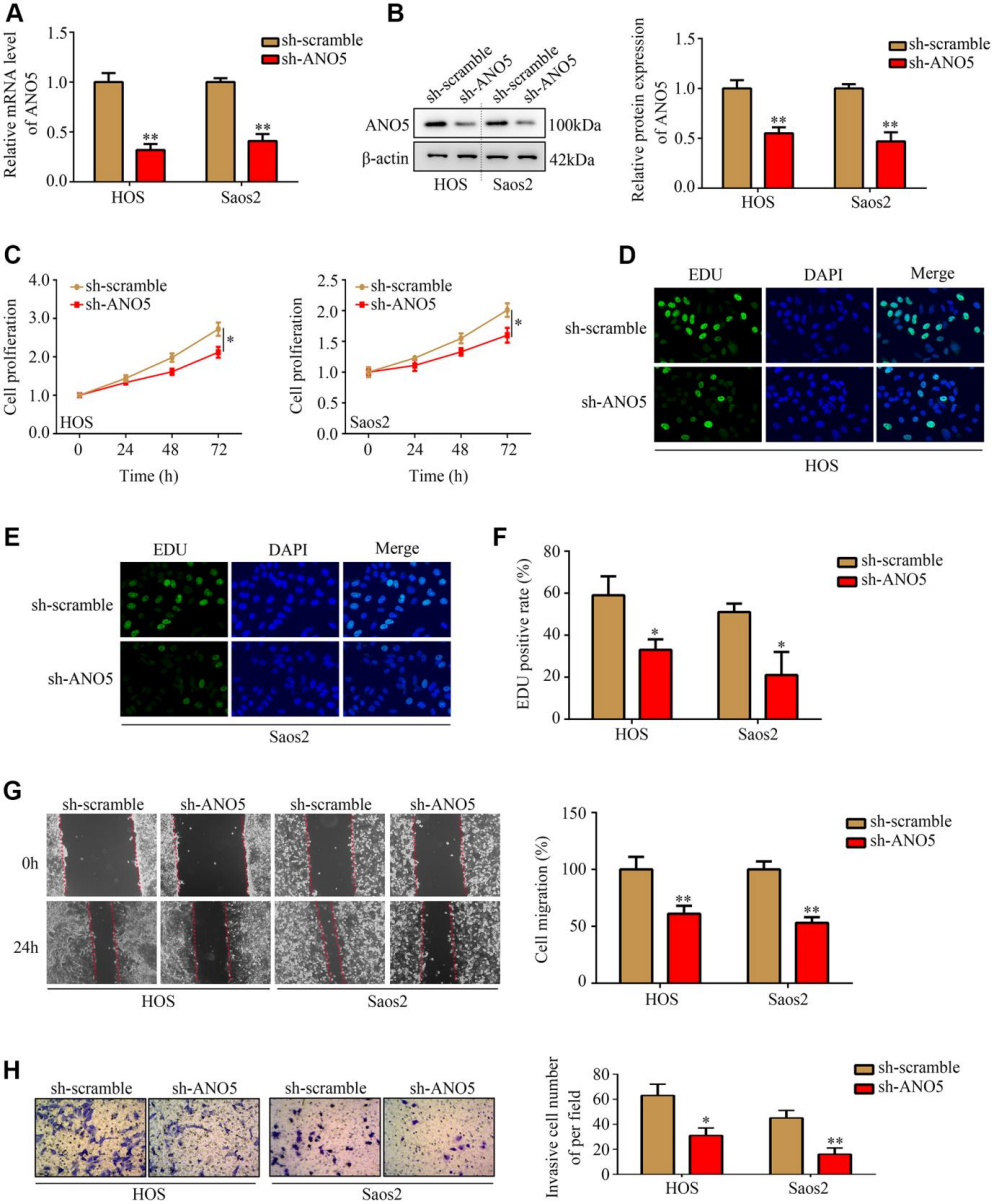
**Inhibition of ANO5 decreased osteosarcoma cell proliferation and metastasis *in vitro*.** (**A**, **B**) qRT-PCR and western blot were used to determine the transfection efficiency of targeted ANO5 shRNA. (**C**) A CCK-8 assay was used to evaluate the effects of ANO5 knockdown on osteosarcoma cell proliferation. (**D**–**F**) EDU assays showed that proliferation rates were reduced in ANO5 knockdown osteosarcoma cells. (**G**) A wound healing assay was used to examine the effect of ANO5 inhibition on osteosarcoma cell migration. (**H**) A transwell assay was used to determine the effect of ANO5 knockdown on osteosarcoma cell invasion. *, *P*<0.05; **, *P*<0.01.

To examine the role of ANO5 in osteosarcoma, short hairpin RNAs were used to established ANO5 knockdown cells (Figure 2A, 2B). CCK-8 and EDU assays showed that inhibition of ANO5 decreased HOS and Saos2 cell proliferation (Figure 2C–2F). A wound healing assay showed that inhibition of ANO5 significantly suppressed the migration of HOS and Saos2 cells (Figure 2G). Moreover, a transwell assay indicated that suppression of ANO5 decreased HOS and Saos2 cell invasion (Figure 2H).

### Overexpression of ANO5 increased osteosarcoma proliferation and metastasis *in vitro*

**Figure 3:**
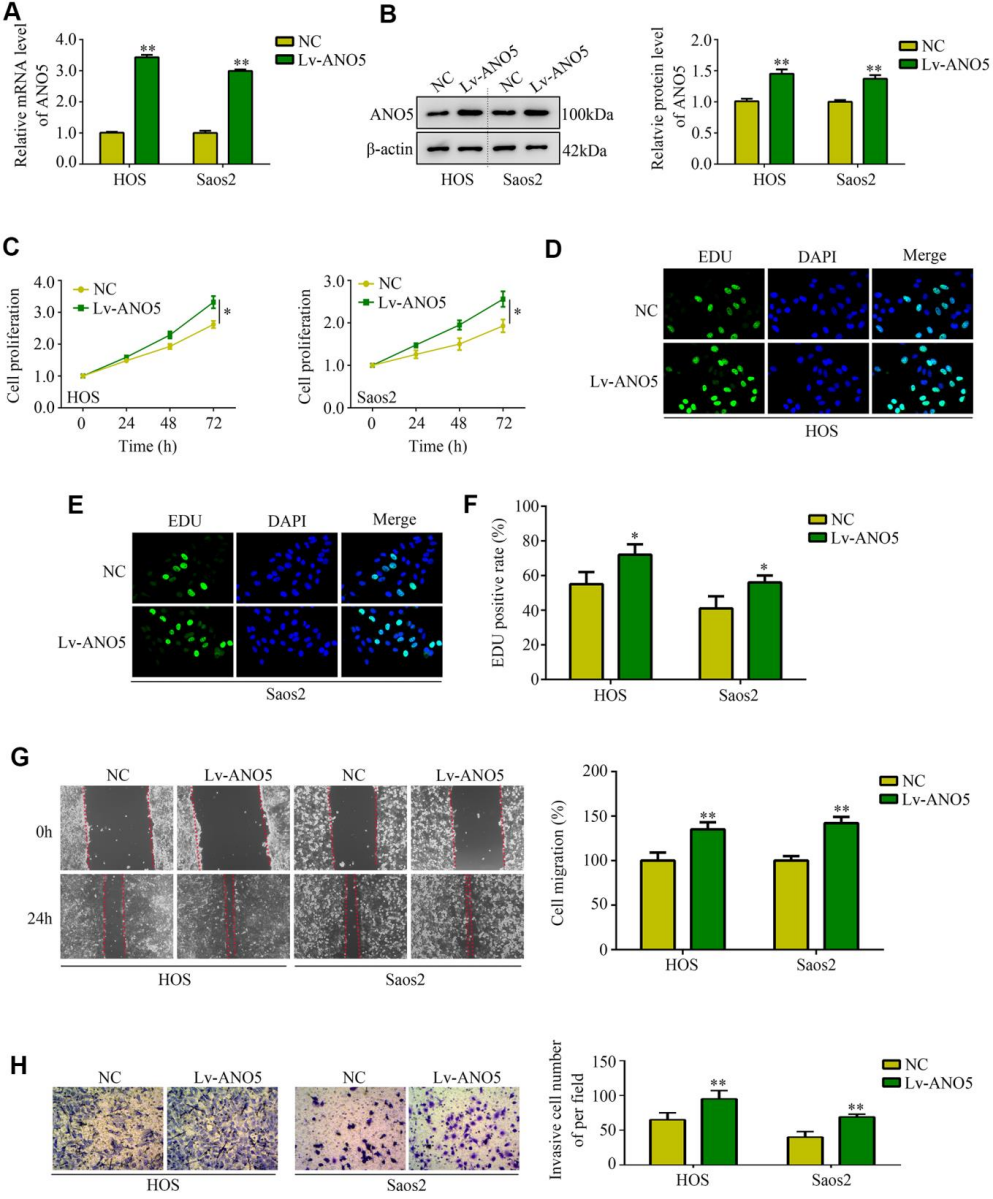
**Overexpression of ANO5 increased osteosarcoma cell proliferation and metastasis *in vitro*.** (**A**, **B**) qRT-PCR and western blot were used to evaluate the transfection efficiency of ANO5 overexpression lentivirus. (**C**) A CCK-8 assay was used to examine the effects of ANO5 overexpression on osteosarcoma cell proliferation. (**D**–**F**) EDU assays showed that proliferation rates were higher in ANO5 overexpression osteosarcoma cells. (**G**) A wound healing assay was used to determine the effect of ANO5 overexpression on osteosarcoma cell migration. (**H**) A transwell assay was used to determine the effect of ANO5 overexpression on osteosarcoma cell invasion. *, *P*<0.05; **, *P*<0.01.

Next, we used an ANO5 overexpression lentivirus to generate ANO5 overexpression cell lines (Figure 3A, 3B). CCK-8 and EDU assays indicated that ANO5 overexpression increased the proliferation rate of HOS and Saos2 cells (Figure 3C–3F). Similarly, wound healing assays demonstrated that overexpression of ANO5 promoted migration in HOS and Saos2 cells (Figure 3G). Furthermore, increased ANO5 expression promoted invasion in HOS and Saos2 cells (Figure 3H). Taken together, these *in vitro* experiments indicated that ANO5 acts as oncogene by promoting osteosarcoma cell proliferation and metastasis.

### ANO5 bound to and increased degradation of NELL1 and NELL2

**Figure 4:**
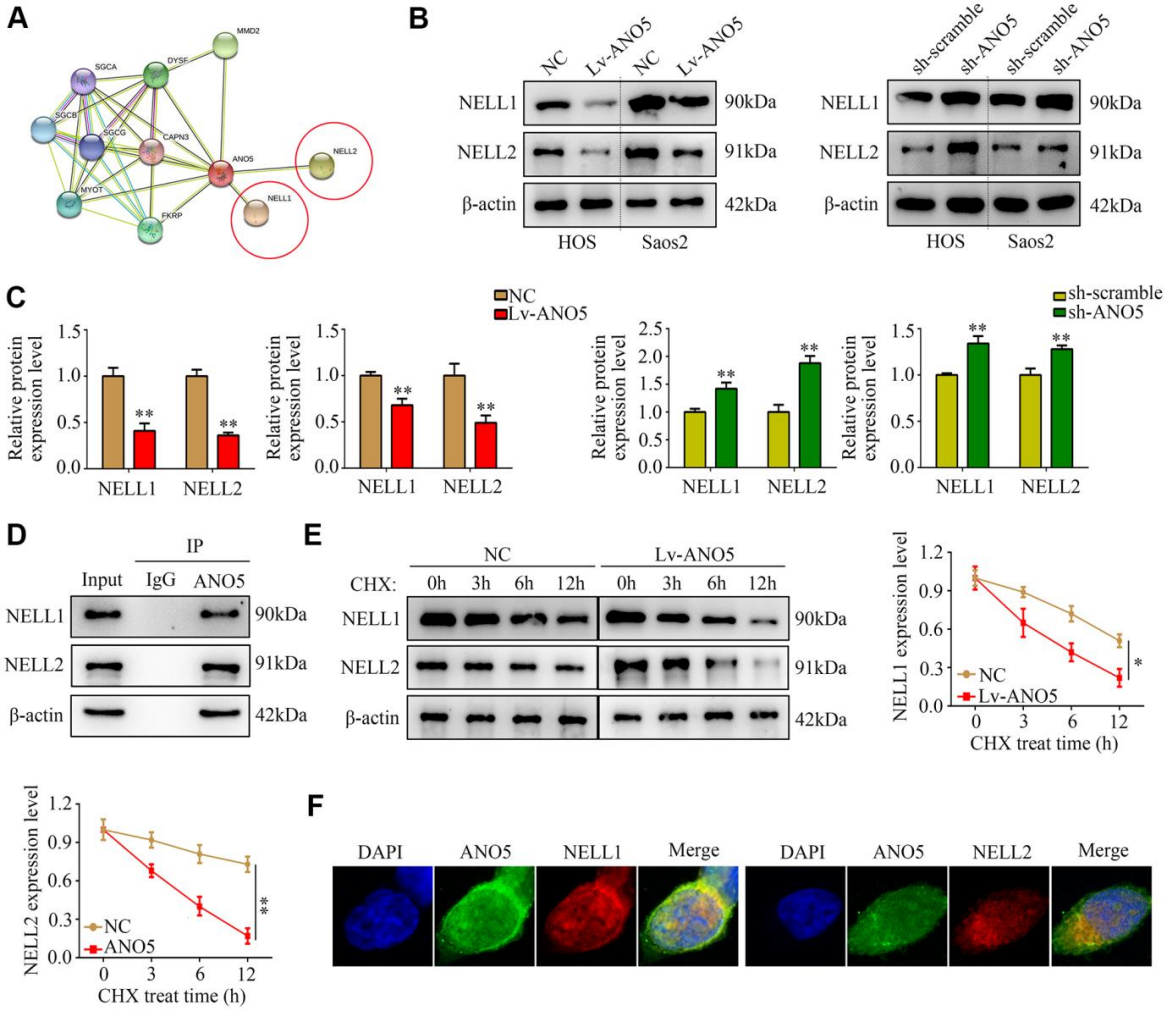
**ANO5 bound to and promoted degradation of NELL1 and NELL2.** (**A**) The protein-protein interaction network showed that ANO5 bound to NELL1 and NELL2. (**B**, **C**) Western blot showed that ANO5 overexpression decreased, while ANO5 knockdown increased, the expression of NELL1 and NELL2. (**D**) Immunoprecipitation demonstrated that ANO5 directly bound with NELL1 and NELL2. (**E**) Overexpression of ANO5 increased the degradation of NELL1 and NELL2. (**F**) ANO5 was colocalized with NELL1 and NELL2 in osteosarcoma cells. *, *P*<0.05; **, *P*<0.01.

To explore how ANO5 promotes osteosarcoma development, the online tool STRING was used to identify proteins that interact with ANO5. NELL1 and NELL2, which act as suppressors in various types of cancer including osteosarcoma, were identified as proteins that interact with ANO5 (Figure 4A). We found that NELL1 and NELL2 protein levels were decreased in ANO5 overexpression cells and were increased in ANO5 knockdown cells (Figure 4B, 4C). In addition, immunoprecipitation demonstrated that ANO5 directly interacted with NELL1 and NELL2 (Figure 4D). Moreover, a cycloheximide (CHX, 10 μM) was used and the result indicated that overexpression of ANO5 increased the degradation rates of NELL1 and NELL2 (Figure 4E). Furthermore, confocal microscopy demonstrated that ANO5 was colocalized with NELL1 and NELL2 in osteosarcoma cells (Figure 4F).

### Suppression of ANO5 decreased osteosarcoma cell proliferation and increased NELL1 and NELL2 expression *in vivo*

**Figure 5:**
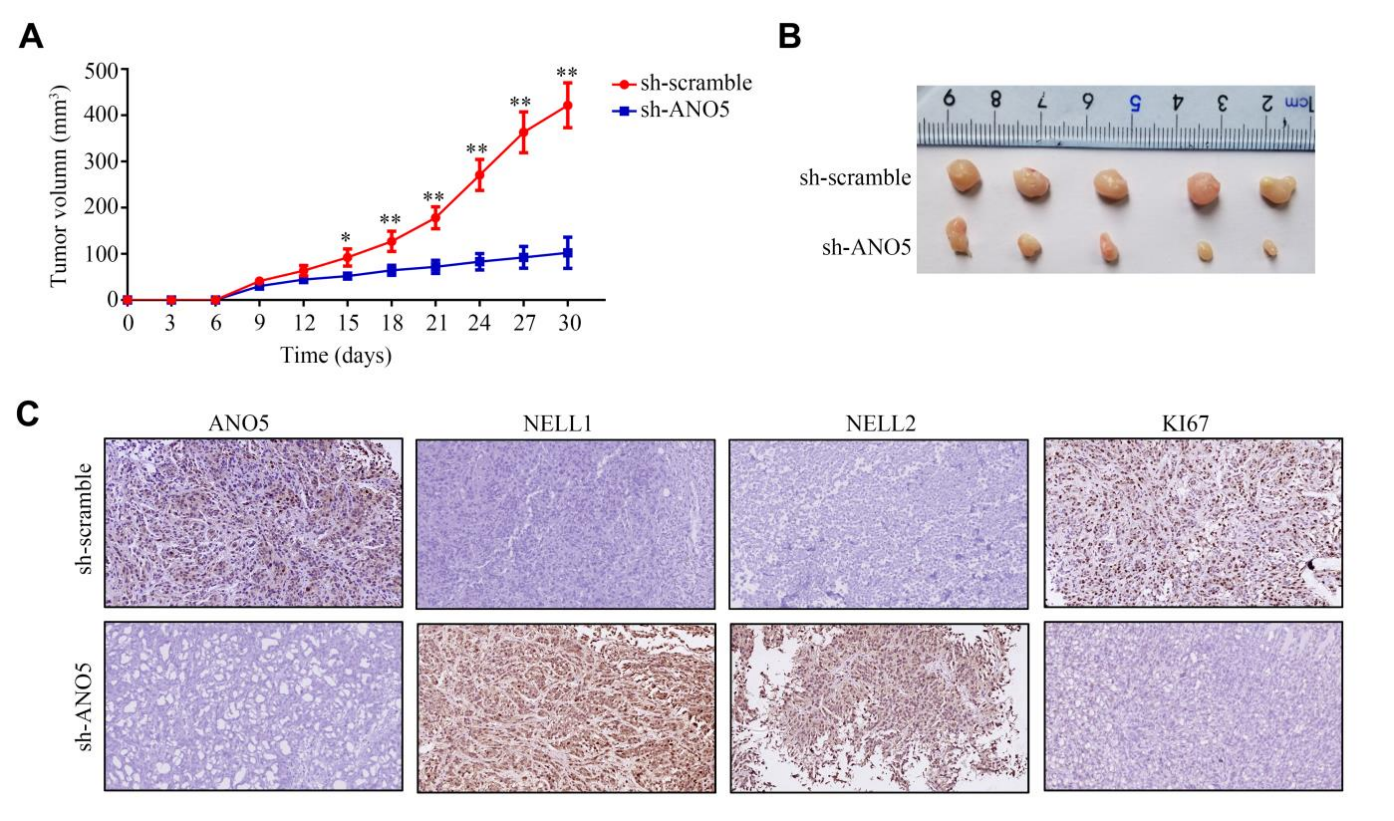
**Inhibition of ANO5 decreased cell proliferation and NELL1 and NELL2 expression *in vivo*.** (**A**, **B**) ANO5 knockdown decreased HOS cell proliferation *in vivo*. (**C**) IHC showed that inhibition of ANO5 decreased the expression of KI67 and increased the expression of NELL1 and NELL2. *, *P*<0.05; **, *P*<0.01.

A subcutaneous tumor transplantation model was then employed to examine the effects of ANO5 on osteosarcoma cell proliferation and NELL1 and NELL2 expression *in vivo*. The results showed that inhibition of ANO5 markedly reduced osteosarcoma cell proliferation rates *in vivo* (Figure 5A, 5B). Furthermore, both NELL1 and NELL2 were increased in tumor tissues with lower ANO5 expression, while the expression of KI67 (a biomarker for proliferation) was decreased (Figure 5C). Taken together, these findings demonstrate that suppression of ANO5 decreased osteosarcoma cell proliferation and increased NELL1 and NELL2 expression *in vivo*.

### Inhibition of NELL1 or NELL2 reversed the inhibitory effects of ANO5 knockdown in osteosarcoma cells

**Figure 6:**
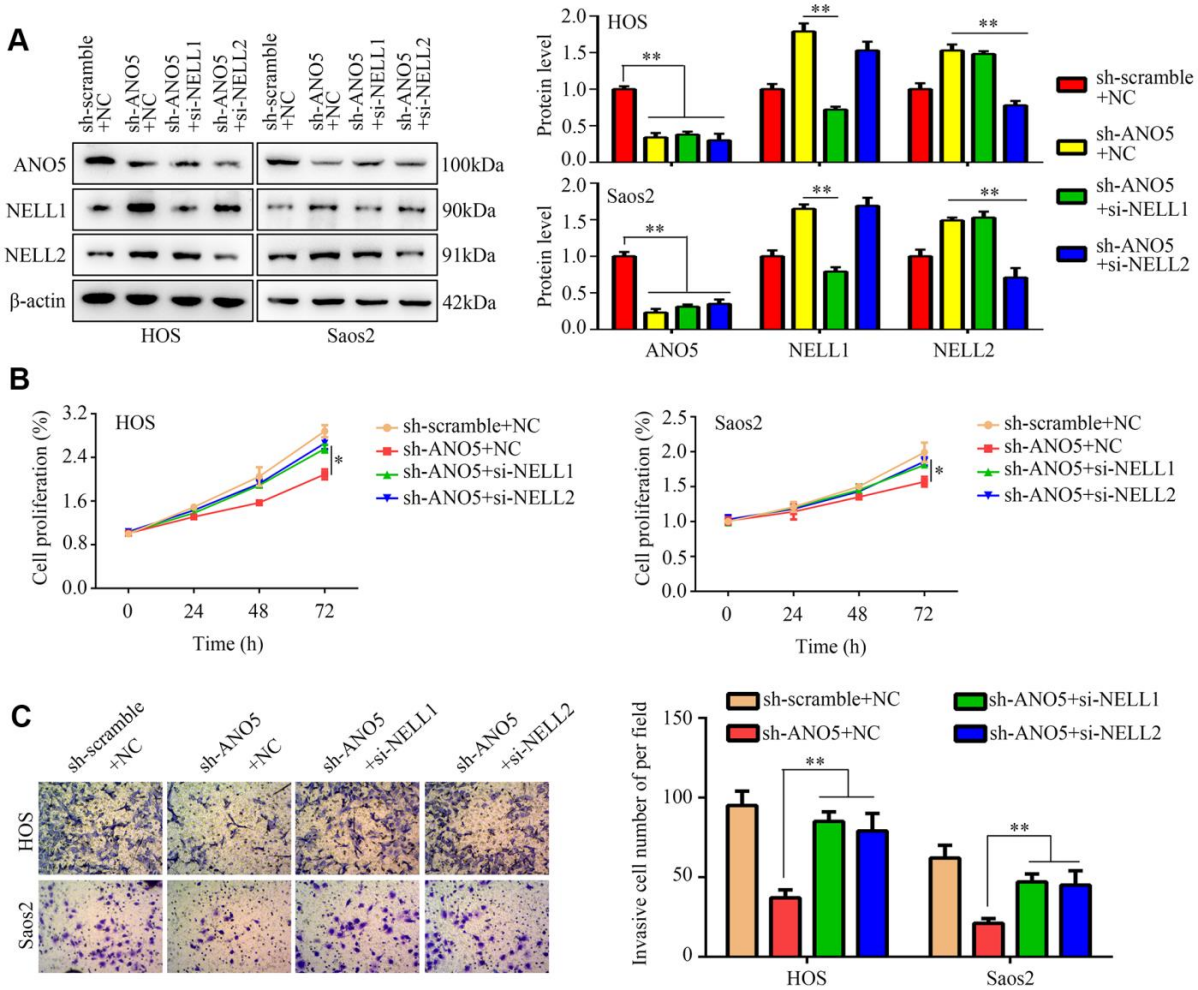
**NELL1 and NELL2 knockdown suppressed ANO5-induced inhibition of proliferation and invasion.** (**A**) Western blot was used to detect the effects of NELL1 and NELL2 inhibition on expression of ANO5, NELL1, and NELL2 in ANO5 knockdown cells. (**B**) CCK-8 was used to detect the effects of NELL1 and NELL2 knockdown on ANO5 suppression-induced inhibition of cell proliferation. (**C**) A transwell assay was used to detect the effects of NELL1 and NELL2 knockdown on ANO5 suppression-induced inhibition of cell invasion. *, *P*<0.05; **, *P*<0.01.

To determine whether NELL1 and NELL2 were involved in ANO5-induced osteosarcoma, we inhibited NELL1 or NELL2 expression in ANO5 knockdown cells (Figure 6A). A CCK-8 assay indicated that suppression of either NELL1 or NELL2 in ANO5 knockdown cells significantly reversed ANO5 suppression-induced inhibition of cell proliferation (Figure 6B). Similarly, ANO5 knockdown cells with NELL1 or NELL2 inhibition showed higher invasive ability than cells without NELL1 or NELL2 inhibition (Figure 6C).

## DISCUSSION

Osteosarcoma is a severe bone tissue malignancy characterized by an extremely poor prognosis. Because of the high efficacy of targeted gene therapy, identification of the molecular mechanisms underlying osteosarcoma progression is particularly important [14]. Here, we explored the biological functions and molecular mechanisms of ANO5 in osteosarcoma. We found that ANO5 promoted the proliferation and metastasis of osteosarcoma by increasing the degradation of NELL1 and NELL2.

Previous studies on the role of ANO5 in cancer development have reported contradictory results. Yu et al. demonstrated that ANO5 was downregulated in prostate cancer and lower expression of ANO5 predicted poorer prognosis [15]. Similarly, Chang et al. found that ANO5 suppressed the mobility of thyroid cancer cells by decreasing JAK/STAT pathway activity [16]. However, Song et al. showed that ANO5 expression was increased in pancreatic cancer and promoted cell proliferation and metastasis [17]. In addition, Ishaque et al. showed that ANO5 expression was positively associated with metastasis of colorectal cancer [18]. In the current study, bioinformatic analysis, western blots, and immunohistochemical staining demonstrated that ANO5 expression was higher in osteosarcoma tissues and cell lines than in adjacent tissues and hFOB1.19 cells. High ANO5 expression was positively associated with tumor size, tumor grade, and metastasis. Furthermore, by performing loss and gain of function experiments, we found that ANO5 promoted proliferation and metastasis of osteosarcoma cells *in vitro*. To our knowledge, these are the first findings to indicate that ANO5 may act as onco-gene in osteosarcoma.

The biological functions of a protein are typically a result of its interactions with other proteins. The STRING online database can predict protein-protein association networks and perform functional enrichment analysis [19, 20]. The STRING interaction network for ANO5 indicated that it interacted with NELL1 and NELL2, suggesting that these proteins may play a role in ANO5-induced proliferation and metastasis in osteosarcoma. In previous studies, NELL1 and NELL2, which act as tumor suppressors, were downregulated in a variety of cancers including osteosarcoma. For example, Peters et al. demonstrated that lower NELL1 expression predicted advanced metastasis in patients with renal cell cancer [21]. Additionally, Maeda et al. showed that both NELL1 and NELL2 were downregulated in glioma [22]. Furthermore, hypermethylation of NELL1 and NELL2, which decreased their transcription, was common in cancer [23]. Here, we found that ANO5 overexpression reduced, while inhibition of ANO5 increased, NELL1 and NELL2 expression. Previous research indicates that proteins can regulate the stability of their binding partner proteins. We found that ANO5 directly bound to NELL1 and NELL2, and ANO5 overexpression increased the degradation of both NELL1 and NELL2. Inhibition of ANO5 increased NELL1 and NELL2 expression *in vivo* and decreased tumor proliferation. Suppression of NELL1 or NELL2 in ANO5 knockdown cells reversed the anti-proliferation effects of ANO5 inhibition. To our knowledge, this is the first evidence that NELL1 and NELL2 are involved in the biological functions of ANO5. We also identified a novel mechanism in addition to DNA hypermethylation by which the tumor suppressors NELL1 and NELL2 were downregulated in cancer tissues.

Taken together, our current results demonstrate that ANO5 acted as a novel oncogene in osteosarcoma. ANO5 expression was elevated in osteosarcoma and promoted the development of osteosarcoma cells by increasing degradation of the tumor suppressors NELL1 and NELL2. ANO5 might therefore be a potential target for osteosarcoma therapy.

## MATERIALS AND METHODS

### Patients and tissue specimens

This study was approved by the Affiliated Hospital of Guizhou Medical University. A total of 40 paired osteosarcoma tissues and adjacent normal tissues were collected from the patients (mean age: 19.43 ± 7.27 years; male/female:27/13) at the pathology department, Affiliated Hospital of Guizhou Medical University, China, between March 2018 and May 2020. The inclusion criteria were as follows: (1) tissues were obtained during surgery and osteosarcoma was diagnosed by two pathologists; (2) patients were diagnosed and treated for the first time; (3) patients agreed to participate in the study. The exclusion criteria were as follows: (1) patients with other malignancies; (2) patients with other systemic diseases; (3) patients who had received treatment prior to admission; (4) patients who did not agree to participate. None of the patients received chemotherapy or radiotherapy before sample collection, and all patients involved in the current study provided written informed consent.

### Bioinformatics analysis

The gene expression profile GSE32395, which contains data from the normal osteoblast cell line hFOB 1.19, the normal bone marrow stromal cell line L87/4, and seven osteosarcoma cell lines (HOS, HOS-58, U2OS, Saos2, MNNG, SJSA, and MG63) was downloaded from GENE EXPRESSION OMNIBUS (GEO, URL: https://www.ncbi.nlm.nih.gov/gds). After standardization, the edgeR package was used to analyze differences in gene expression in R software (Version: 4.0.2); the threshold was set at LogFC>2, and an adjusted P value<0.05 was considered significant. Differentially expressed genes were shown in a volcano plot. The STRING website (URL: https://string-db.org/ was used to identify proteins that interact with ANO5 using a protein-protein interaction network.

### Cell culture and transfection

The normal osteoblast cell line hFOB 1.19 and four osteosarcoma cell lines (U2OS, MG63, HOS, and Saos2) were obtained from ATCC (USA). All cells were cultured in Dulbecco's modified Eagle's medium (DMEM; Boster, Wuhan, China) containing 10% fetal bovine serum (FBS; Hyclone, USA) at 37° C in an environment with 5% CO_2_. ANO5 overexpression lentiviruses, negative control lentiviruses (NC), short hairpin RNA (shRNA) against ANO5 (sh-ANO5), and scramble shRNA (sh-scramble) were purchased from Genechem (Shanghai, China). Small interfering RNA (siRNA) targeting NELL1 and NELL2 and corresponding negative control siRNAs were purchased from GeneCopoeia (Guangzhou, China). The sequences were as follows: sh-ANO5, GCUGUAGUUGGCUUAGCUUTT; sh-scramble, UUCUCCGAACGUGUCACGUTT; NELL1 siRNA, GAGCCTGGTTCAAGGAATA; NELL2 siRNA, GGACGAAAGCCUUCCUCUU. Transfection of lentiviruses, shRNAs, and siRNAs was performed using Lipo2000 (Invitrogen, USA) according to the manufacturer's instruction. To promote stable expression, cells were then incubated continuously with 1 μg/mL puromycin for 12 days.

### qRT-PCR

Total RNA was isolated from osteosarcoma tissues and cells using TRIzol (Takara, Japan). cDNA was synthesized from 2 μg isolated mRNA using Hifair^®^ II 1st Strand cDNA Synthesis SuperMix (Yeasen, Shanghai, China). The Hifair^®^ III One Step RT-qPCR SYBR Green Kit (Yeasen, Shanghai, China) was used for qPCR. The qPCR reaction system included the following: 1 μL cDNA template, 0.25 μL forward primer, 0.25 μL reverse primer, 6.75 μL SYBR Green reagent, and 4.25 μL dH_2_O. The qPCR program consisted of 5 min at 95° C and 35 cycles of 30 seconds at 95° C, 30 seconds at 65° C, and 1 minute at 70° C. β-actin was used as the reference gene for determining relative expression of the target gene. ANO5 and β-actin primers were as follows: ANO5 forward primer, 5’-GCGGCGGCTTATGTTTCAAAA-3’; ANO5 reverse primer, 5’-CGCCTTTAACTCTGCGTCTTTC-3’; β-actin forward primer, 5’-CATGTACGTTGCTATCCAGGC-3’; β-actin reverse primer, 5’-CTCCTTAATGTCACGCAC GAT-3’.

### Western blot

Whole protein was isolated from osteosarcoma cells using RIPA reagent (Boster, Wuhan, China) containing 10% PMSF inhibitor. Protein samples were separated using 10% SDS-PAGE at 100 V for 2 h and transferred into PVDF membranes at 310 mA for 2 h. After blocking with 10% non-fat milk for 2 h, primary antibodies against ANO5 (dilution 1:1000; cat no. PA5-109393, Sigma, USA), NELL1 (dilution 1:500; cat no. PA5-27958, Sigma, USA), NELL2 (dilution 1:500; cat no. PA5-42910, Sigma, USA), and β-actin (dilution 1:2000; cat no. 20536-1-AP, Proteintech, Wuhan, China) were added and incubated at 4° C overnight. The membranes were then incubated with secondary antibodies after washing three times with TBST. Finally, ECL reagent was used to visualize the bands, and β-actin was used as a loading control to calculate relative expression of target proteins.

### Immunohistochemistry (IHC)

Osteosarcoma tissues and adjacent tissues were sliced into 2 μm thick sections and then incubated at 65° C for 2 h. The sections were then dewaxed in xylene and rehydrated via an ethyl alcohol concentration gradient. Antigen retrieval was performed via a high-pressure method with an EDTA reagent (Boster, Wuhan, China). After blocking using H_2_O_2_ and 5% BSA, primary antibodies against ANO5 (dilution 1:200; cat no. PA5-63619, Sigma, USA), NELL1 (dilution 1:200; cat no. PA5-27958, Sigma, USA), NELL2 (dilution 1:200; cat no. PA5-57373, Sigma, USA), and KI67 (dilution 1:200; cat no. PA5-19462, Sigma, USA) were added and incubated at 4° C overnight. Then, secondary antibodies were added and incubated at room temperature for 2 h. Finally, after staining with DAB (Servicebio, Wuhan, China), hematoxylin was used to stain cell nuclei and images were obtained using a light microscope (200×, Nikon, Japan).

### Cell count kit-8 (CCK-8) assay

A total of 5×10^3^ HOS and Saos2 cells were plated into the wells of a 96 well-plate. After 24 h, 48 h, or 72 h, 10 μL of CCK-8 reagent (Boster, Wuhan, China) was added to each well. After culturing for an additional 2 h at 37° C, the absorbance of each well at 450 nm was measured.

### 5-Ethynyl-2 (EDU) assay

Osteosarcoma cells were added to 6-well plates (5×10^5^ cells/well) with DMEM (10% FBS) and cultured for 24 h. Then, the cells were incubated with 50 μM EdU reagent (Beyotime, Suzhou, China) at 37° C for 2.5 h, fixed with 4% paraformaldehyde (Servicebio, Wuhan, China) and 0.5% Triton reagent (Servicebio, Wuhan, China) for 30 min, and stained with 1× Apollo^®^ reaction cocktail (Beyotime, Suzhou, China) for 40 min. Finally, cell nuclei was stained with DAPI (Servicebio, Wuhan, China), and cells were visualized using a fluorescence microscope (Nikon, Japan).

### Wound healing assay

A total of 5×10^5^ osteosarcoma cells were plated in 6-well plates and allowed to reach 95% confluence. Then, a 200 μL pipette tip was used to form a wound by scraping. After washing with PBS three times to remove floating cells, wound healing was assessed at 0 h and 24 h using a light microscope (40×). Migration ability was calculated according to the following formula: cell migration (%) = (area in treatment group at 0 h – area in treatment group at 24h) / (area in control group at 0 h – area in control group at 24 h) × 100%.

### Transwell assay

A total of 2×10^4^ osteosarcoma cells were plated in the upper chambers of a transwell plate (0.8μm, Corning, USA) containing Matrigel (Corning, USA) with 300 μL of FBS-free DMEM medium. Then, 700 μL of DMEM medium containing 10% FBS was placed in the lower transwell chamber as a chemoattractant. After culturing for 24 h, the transwells were fixed, stained with 1% crystal violet for 30 min, and visualized using a light microscope (100×). The invasive ability of cells was evaluated by the average number of invaded cells in five random fields.

### Immunoprecipitation

Cells were lysed in weak RIPA buffer (Boster, Wuhan, China) containing 1% PMSF. The liquid was centrifuged and the protein was collected. Then, anti-ANO5 (dilution 1:50) antibody and IgG (dilution 1:50; Beyotime; Hangzhou, China) were added for 6 h. A/G-agarose beads (MCE, Wuhan, China) were then added and incubated for 3h. After washing three times with PBS, isolated immunoprecipitates attached to the beads were collected and analyzed using western blot.

### Confocal microscopy

Osteosarcoma cells were injected into a confocal dish (5×10^5^ cells/well) with DMEM (10% FBS) and cultured for 24 h. Then, the cells were fixed and treated with 0.5% Triton reagent for 8 min at room temperature. After blocking with 5% BSA, the cells were incubated with anti-ANO5, anti-NELL1, and anti-NELL2 antibodies at 4° C overnight. After incubating with secondary antibodies, nuclei were stained with DAPI (Servicebio, Wuhan, China), and the cells were visualized using a confocal microscope (Nikon, Japan).

### Animal experiments

Ten female BALB/c nude mice (4-6 weeks old; weight 16-18 g) were obtained from Beijing Huafukang Biotech (URL: http://www.hfkbio.com/). The mice were housed at 23–24° C, and the light-dark cycle was set at 12 h intervals. A total of 5 ×10^6^ HOS cells with ANO5 inhibition or NC HOS cells were injected subcutaneously into the right armpits of the mice (n=5 mice per group). Tumor proliferation was evaluated every three days, and the formula for calculating tumor volume was (length×width^2^)/2. After 30 days, the mice were sacrificed, and tumors were dissected to perform IHC. The animal experiments in this study were approved by the Animal Ethics Committee of Guizhou Medical University.

### Data analysis

All data were analyzed using SPSS 20.0 software (IBM Corp., USA). ANOVA with Bonferroni’s post hoc test was used to analyze differences between multiple groups. T-tests were used to examine differences between two groups. *P*<0.05 was set as the cut-off for statistical significance.

### Data availability

Data collected in this study are available from the corresponding author upon request.
